# Microbiota-gut-liver-brain axis and hepatic encephalopathy

**DOI:** 10.20517/mrr.2023.44

**Published:** 2024-01-25

**Authors:** Haifeng Lu, Hua Zhang, Zhongwen Wu, Lanjuan Li

**Affiliations:** State Key Laboratory for Diagnosis and Treatment of Infectious Diseases, National Clinical Research Center for Infectious Diseases, National Medical Center for Infectious Diseases, Collaborative Innovation Center for Diagnosis and Treatment of Infectious Diseases, The First Affiliated Hospital, Zhejiang University School of Medicine, Hangzhou 310003, Zhejiang, China.

**Keywords:** Hepatic encephalopathy, hyperammonemia, gut microbiota-liver-brain axis, microbiome-targeted therapy

## Abstract

Hepatic encephalopathy (HE) is a clinical manifestation of neurological and psychiatric abnormalities that are caused by complications of liver dysfunction including hyperammonemia, hyperuricemia, and portal hypertension. Accumulating evidence suggests that HE could be reversed through therapeutic modifications of gut microbiota. Multiple preclinical and clinical studies have indicated that gut microbiome affects the physiological function of the liver, such as the regulation of metabolism, secretion, and immunity, through the gut-liver crosstalk. In addition, gut microbiota also influences the brain through the gut-brain crosstalk, altering its physiological functions including the regulation of the immune, neuroendocrine, and vagal pathways. Thus, key molecules that are involved in the microbiota-gut-liver-brain axis might be able to serve as clinical biomarkers for early diagnosis of HE, and could be effective therapeutic targets for clinical interventions. In this review, we summarize the pathophysiology of HE and further propose approaches modulating the microbiota-gut-liver-brain axis in order to provide a comprehensive understanding of the prevention and potential clinical treatment for HE with a microbiota-targeted therapy.

## INTRODUCTION

Hepatic encephalopathy (HE) is a clinical manifestation of neurological and psychiatric abnormalities caused by complications stemming from liver dysfunction, including hyperammonemia and portal hypertension^[1]^. As the disease progresses, many patients experience neurological symptoms including sleep disorders, personality changes, depression, coma, and even death. Once HE is developed in patients with liver disease, it takes a long time for them to recover cognitive function, even with successful treatments. Additionally, secondary HE may recur during the recovery period, which can seriously affect the prognosis and quality of life of the patients^[2]^. Various factors that cause acute liver failure and cirrhosis can induce HE, for example, hepatitis viruses such as HBV, HCV, HEV, drugs, or liver toxic substances such as alcohol, chemical agents, *etc*. Generally, some neurotoxic molecules should be effectively metabolized while passing through the liver before entering the systemic circulation. Otherwise, it will result in pathological neurological disorder syndromes. Additionally, abnormal functioning of the urea cycle enzymes or any other factors, for example, congenital urea cycle disorders, can lead to elevated blood ammonia (hyperammonemia), which is believed to be a major contributor to the neurological sequelae following severe liver disease. Depending on the underlying liver disease, HE is divided into three types: Type A, caused by acute liver failure, accompanied with relatively minor changes in consciousness at first, and then rapidly falling into deep coma and even death; Type B, caused by portocaval shunting, without any apparent liver disease; Type C, caused by liver cirrhosis, characterized by chronic recurrent personality and behavioral changes, cognitive impairment, and even numbness and coma. HE is a continuous process constantly changing from normal cognitive function, clear consciousness, to coma^[3]^. Clinical treatment mainly focuses on supportive therapies and the treatment of symptoms. HE has become an urgent issue for medical research due to the poor effectiveness of treatment and high mortality rate. Currently, early prediction, early identification, and timely treatment are highly effective strategies to prevent and treat HE. Reports indicate that early diagnosis and treatment for patients at high risk of HE is associated with improvement in the prognosis of HE^[4]^.

The close connection between the physiological functions of the gut-liver axis^[5]^ and the gut-brain axis^[6]^, both of which are in part mediated by gut microbiota, has become an increasingly important topic for HE medical research. Although HE is caused by liver failure, accumulating evidence suggests that there are bidirectional links among the intestine, the liver, and the brain, known as the gut-liver-brain axis, which involves the exchange of information via hormones, cytokines, and nutritional metabolites^[7,8]^. Most HE patients display an increased permeability of the blood-cerebrospinal fluid barrier, which renders their brains vulnerable to toxins and the toxic effects of neurotoxins. In addition, artificial liver support for HE patients has been shown to improve the prognosis, shorten disease course, and reduce mortality^[9]^.

The human gastrointestinal tract harbors a wide variety of microbes, the so-called gut microbiome - the second genome^[10,11]^. From birth, the coevolution of gut microbiota and the host shapes a unique pattern of gene expression and regulation, which has become the focus of precision medicine research in the 21st century. Gut microbiota is involved in the digestion and metabolism of nutrients and various drugs, and also regulates immune responses^[12-14]^. Furthermore, it makes an important contribution to the regulation of development, maturation, and physiological functions of the gut, liver, and central nervous system^[15]^. Since gut microbiota has been shown to be involved in regulating central nervous system activities such as brain development, stress response, anxiety, depression, and cognitive function^[16]^, further exploration of the molecular interaction between the intestinal microbiome and the gut-liver-brain axis is of great significance for the clinical prevention, treatment, and prognosis of HE. This review focuses on the communication network between gut microbiota and the gut-liver-brain axis, which is referred to as the gut microbiota-liver-brain axis, and the role and significance of this network in the occurrence, prognosis, prevention, and treatment of HE.

## THE MICROBIOME-GUT-LIVER-BRAIN AXIS: CONNECTING THE GUT MICROBIOME AND HEPATIC ENCEPHALOPATHY

**Figure 1:**
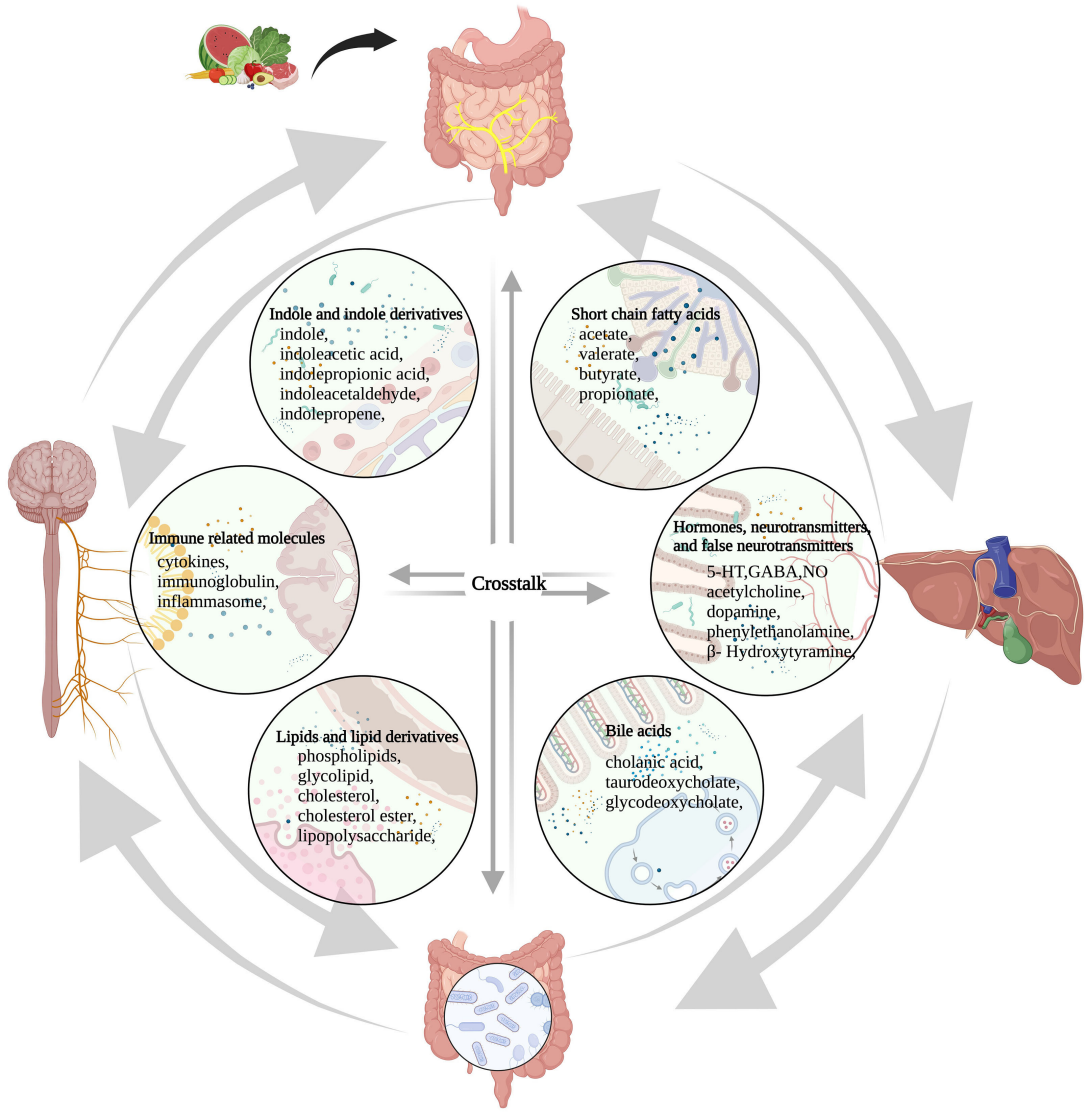
The microbiome-gut-liver-brain axis and molecules involved in this network. These molecules are classified into six categories, including short-chain fatty acids, hormones, neurotransmitters and false neurotransmitters, bile acids, lipids and lipid derivatives, and immune-related molecules, indole and indole derivatives. Amino acids, glucose, protein, fatty acids, vitamins, minerals, and other nutrients that are absorbed from food in the gut participate in the synthesis and conversion of these molecules directly or indirectly. 5-HT: 5-hydroxytryptamine; GABA: γ-aminobutyric acid; NO: nitric oxide.

The microbiota-gut-brain axis is a communication network connecting the gut and the brain structurally and functionally, and is operated through bioactive molecules, the vagus nerve, and the neuroendocrine and immune pathway [Figure 1]^[17]^. The intestinal microbiome plays a role in basic neurogenesis, such as the formation of the blood-brain barrier, generation of myelin sheath, neurogenesis, and microglia maturation^[18]^. It also participates in regulating various behaviors^[19]^. Recent studies have indicated that dysbiosis of the gut microbiome is an important environmental factor that contributes to the induction and development of nervous system dysfunction such as mental disease^[20]^, central nervous system degeneration^[21,22]^, and irritable bowel syndrome^[23]^. Evidence suggests an association between the gut microbiota and brain regions controlling sensory information^[24]^. In particular, signals generated by the brain can shape the composition of gut microbiota, and molecules produced by the gut microbiome can affect the structure of the human brain, especially for false neurotransmitters. Therefore, neurotransmitters produced from the brain and false neurotransmitters produced from the gut microbiome, through their action on the gut and brain, respectively, play a critical role in the communication between the microbiome, the gut, and the brain^[25]^. A comprehensive understanding of the interaction between the gut microbiome and the brain will provide us with new ideas for the development of clinical strategies to prevent and treat HE^[26]^. Additionally, a mutual communication exists between the liver and the gut through the bile duct, portal vein, and systemic circulation. Liver metabolites affect the gut microbiome and the function of gut barrier, and conversely, the gut microbiome participates in regulating bile acid synthesis and glycolipid metabolism in the liver^[5]^. Proinflammatory factors in the liver and intestines mediate the development of liver fibrosis, cirrhosis, and hepatocellular carcinoma^[27]^. Many studies on the pathogenesis of liver disease confirmed the close relationship between intestinal dysbiosis and the development of liver disease^[26,28]^.

The gut microbiota is involved in the pathogenesis of HE through their participation not only in the gut-liver axis, but also in the gut-brain axis. The gut-liver-brain axis was first proposed in the regulation of liver glycogen metabolism and energy homeostasis^[8]^. It is well-known that the vagus nerve plays a critical role in communication among the gut, the liver, and the brain, as the hepatic vagal sensory afferent nerves link the gut microenvironment to neuronal activity in the nucleus tractus solitarius^[29]^. In addition, the gut-liver-brain axis is involved in a number of processes including immune homeostasis. For example, it regulates the differentiation of peripheral regulatory T cells, and affects the expression of aldehyde dehydrogenase in the intestinal antigen-presenting cells and the synthesis of retinoic acid^[30]^. Another significance of the gut-liver-brain axis is found in the levels of N-acetyl aspartate and brain functions. N-acetyl aspartate is considered a marker of brain dysfunction, and the level of this molecule may indicate the severity of neuronal damage^[31,32]^, although it is synthesized by a mitochondrial enzyme expressed in the liver and kidney^[33]^. Interestingly, the levels of N-acetyl aspartate negatively correlate with the amounts of *Ruminococcus* in the intestine, while they positively correlate with *Butyricicoccus*^[34]^. Finally, the gut-liver-brain axis is also involved in the homeostasis of nitric oxide (NO), which is related to gut-derived bacterial translocation and portal hypertension in cirrhotic patients. Gut microbiome dysbiosis bidirectionally influences the liver microenvironment and is associated with endotoxemia that causes the overproduction of NO through an induction of NO synthase. NO is thought to act as a signaling molecule in the dorsal vagal complex and contributes to autonomic reflex function^[35]^ and peripheral dilatation, which results in portal hypertension in cirrhotic patients. Therefore, systemic NO overproduction and hepatic NO underproduction are factors that contribute to arterial vasodilatation and portal hypertension^[36]^. It is also notable that serum NO level is a biomarker associated with disease severity^[37]^. Importantly, changes in the abundance of selected gut microbiota that have a nitrogen fixation capacity, including *Lactobacteria*, *Bifidobacteria*, *E. coli*, *Klebsiella*, and *Clostridiales* strains, can stimulate expression of inducible NO synthase in host epithelial cells by regulating microglia maturation and activation^[38]^.

Considering the important roles of the gut microbiome in the pathogenesis of liver and brain disease, the molecular network consisting of the microbiome-gut-liver-brain axis should not be neglected in studies on the pathogenesis and treatment of HE. For example, urease-containing bacteria such as *Klebsiella*, *Proteus*, and *Alcaligenes*, which are enriched in the gut microbiome of HE patients, produce neurotoxic substances such as ammonia, mercaptan, benzodiazepine-like compounds^[39]^, manganese, and lipopolysaccharide (LPS) to levels exceeding the metabolic capacity of the liver in HE patients^[40,41]^. Subsequently, these pathogenic conditions trigger the production of other metabolites and immune responses, which reciprocally alter the gut microbiome^[42]^. Ammonia is easily absorbed through the lipid membrane and enters the brain through the blood-brain barrier, causing an impairment of the structure and function of the brain to contribute to the pathogenesis of HE^[43]^. Current clinical therapies targeting gut microbiota, such as the antibiotics rifaximin and lactulose, can reduce blood ammonia and improve cognitive function in HE patients. Furthermore, fecal microbiota transplantation (FMT) has also been shown to improve cognitive function and reduce the recurrence rate of HE in patients^[26]^.

## BIOACTIVE MOLECULES AS MEDIATORS OF THE MICROBIOME-GUT-LIVER-BRAIN AXIS

Although HE is mainly caused by disorders of the liver, dysfunction of the gut microbiome, gut, brain, and immune system is also involved in the disease progression. The gut microbiome is proposed to be an endocrine organ that produces bioactive molecules that can interact with the host’s physiological functions to trigger responses from the gut as well as other distant organs^[44,45]^. These molecules include a large number of metabolites, such as various amino acid metabolites, short-chain fatty acids (SCFAs), secondary bile acids (BAs), and lipids and lipid derivatives.

### Amino acid metabolites

Intestinal bacteria directly or indirectly participate in tryptophan (Trp) metabolism, producing a series of indole derivatives that are considered to be an important mediator for communication and exchange of information between the microbiota and their host^[46,47]^. Some species of gut microbiota (for example, *Clostridium*) directly metabolize Trp to bioactive substances, such as indole, indole acetic acid, indole propionic acid, indole acetaldehyde, and indole propene. These substances can act as ligands for aromatic hydrocarbon receptors and play an important role in immune homeostasis. Moreover, they are involved in the production of neurotransmitters such as 5-hydroxytryptamine (5-HT) that mediate the function of the central nervous system. Intestinal bacterial amino acid decarboxylase can transform phenylalanine, tyrosine, lysine, and glutamic acid into neurotransmitters such as dopamine, acetylcholine, and γ-aminobutyric acid (GABA)^[48]^. GABA is implicated in the pathophysiology and clinical manifestations of neuropsychiatric disorder in most HE patients. Research found that, in the early stage of HE, a high level of plasma ammonia stimulates the consumption of glutamic acid to generate glutamine, which results in decreased GABA synthesis. However, in the late stage, high plasma ammonia inhibits the hydrolysis of GABA, leading to an accumulation of GABA in the brain. Thus, most HE patients exhibit clinical manifestations of anxiety, insomnia, silent excitement and excitement in the early stage, but drowsiness and coma in the late stage. Additionally, these aromatic amines can directly enter the brain when liver function is impaired, and generate pseudo-neurotransmitters including phenylethanolamine and β-hydroxytyramine. These pseudo-neurotransmitters cannot transmit nerve impulses^[49,50]^ and, therefore, may cause brain dysfunction and liver coma. Research on the influence of gut microbiota-derived metabolites on the pathophysiological mechanisms of HE found that hyperammonemia plays an important role in the progression of HE. At present, it is generally believed that ammonia diffuses into the brain through the blood-brain barrier. In the brain, glutamine synthetase in astrocyte cells can catalyze the reaction between glutamate and free ammonia to synthetize glutamine^[43,51]^, which is critical for detoxification of ammonia. In pathological conditions, the capacity of astrocytic detoxification is weakened, and accordingly, the concentrations of brain ammonia are increased. This is followed by astrocyte swelling and decreased nutrient availability and glutamate uptake, which produces continuous stimulation of neurons^[52]^. In addition, because the ability of the liver to convert ammonia to urea is not sufficient in HE patients, making it difficult to keep the blood ammonia concentration at a safe level, the patients are caught in a vicious cycle that exacerbates acute brain damage and liver injury. Gut alkaline protease-producing bacteria, for example, *Enterobacteriaceae* that is increased in HE patients^[40]^, also metabolize proteins, urea, and glutamine, which are the main sources of blood ammonia production.

### Short-chain fatty acids

SCFAs are intestinal microbiota-derived metabolites, and have been reported to be decreased in cirrhotic patients, including those with HE^[53,54]^. SCFAs have been reported to accelerate the release of hormones from intestinal endocrine cells, such as serotonin from intestinal chromaffin cells, which can regulate emotional activity in a paracrine manner^[55]^. A study in mice showed that *B. dentium* and the SCFA acetate can regulate key components of the serotonergic system to influence host behavior by increasing both the concentration of 5-HT and the expression of its receptors, 4- and 5-HT transporters, in the intestine^[56]^. High levels of LPS and certain SCFAs, the acetate and valerate, in the blood were associated with increased deposition of amyloid protein in the brain, which results in Alzheimer’s disease. On the contrary, high levels of another type of SCFA, butyrate, were negatively associated with amyloidosis^[57]^. Butyrate supplementation could improve glucose homeostasis and beta-cell function in mice, which may reduce hepatic lipid accumulation and inflammation^[58]^. In addition, acetate, propionate, and butyrate reduced the production of proinflammatory factors and NO, and inhibited inducible NO synthase, which is involved in the gut-liver-brain axis^[59]^. It has been shown that the abundance of gut SCFA-producing bacteria, such as *Lachnospiraceae*, *Ruminococcaceae*, and *Clostridiales XIV*, was decreased in cirrhotic patients, especially in patients with HE^[40]^.

### Bile acids

BAs, a cluster of hydroxyl derivatives of cholanic acid, are critical to the microbiome-gut-liver-brain axis due to their various physiological functions^[60]^. Primary BAs are synthesized in the liver, discharged into the intestine through the bile duct, and transformed into secondary BAs by enzymes produced by gut bacteria^[61]^. The synthesis, secretion, transformation, reabsorption, and processing of BAs are closely related to the function of the host liver, gallbladder, intestine, and brain^[62]^. The abnormal metabolism of BAs and an imbalance of cholesterol metabolism inevitably affect the function of these organs because of their influence on body energy metabolism and immunity. BA metabolism is mostly mediated by a nuclear receptor Farnesoid X Receptor (FXR), the Takeda G-protein-coupled receptor 5 (TGR5), and sphingosine 1-phosphate receptor 2. TGR5 is involved in the regulation of energy metabolism^[63]^ and activation of the immune response^[64]^. Activation of the TGR5 signaling pathway can increase the expression of several mitochondrial genes involved in energy consumption, trigger an induction in deiodinase 2 genes, and promote the self-phosphorylation of the Sarcoma gene coding (SRC) protein-tyrosine kinases via the TGR5-β-Arrestin-SRC pathway^[65]^. Through phosphorylation, SRC activates various downstream immune response-related proteins, including retinoic acid-inducible gene 1 (RIG-1) protein, virus-induced signaling adapter (VISA) protein, stimulator of interferon gene protein (STING), TANK binding kinase 1 (TBK1) gene protein, and interferon regulatory factor 3 (IRF-3). Binding of sphingosine 1-phosphate receptor 2 by BAs can activate the extracellular signal-regulated kinase (ERK)1/2 and Akt-dependent pathways^[66]^, which subsequently affects blood-brain barrier permeability and may cause brain impairment by promoting neuroinflammation associated with interleukin-1β and tumor necrosis factor-α^[67]^. Aberrant BA signaling in the gut-liver-brain axis may also be an important factor in the pathogenesis of neurodegenerative diseases^[68]^. Conjugated 12α-hydroxylated BAs (taurodeoxycholate and glycodeoxycholate) are reported to have the ability to induce liver fibrogenesis via the ERK1/2 and p38 mitogen-activated protein kinases (MAPK) signaling pathways^[69]^. Microbiota dysbiosis and aberrant BA metabolism have been observed in HE patients^[70,71]^.

### Lipids and lipid derivatives

Lipids include fat, phospholipids, glycolipids, cholesterol, and cholesterol esters, and most of them are transformed in the liver^[72]^. Lipids and lipid derivatives are involved in the supply of energy and essential fatty acids. They also play essential roles in the absorption of fat-soluble vitamins and the metabolism of calcium and phosphorus. In addition, they form a hydrophobic barrier to maintain the normal structure and function of cells. Animal studies suggest that the brain can perceive the levels of lipids in the blood. In addition, lipids in the upper intestine increase the level of long-chain fatty acyl-coenzyme A (LCFA CoA) in the small intestine, and both can inhibit the production of glucose in the liver through the gut-liver-brain axis^[8]^. In other words, lipids absorbed in the upper intestine can inhibit glucose production in the liver through LCFA CoA as a mediator. LCFA CoA is also an important backbone for many bioactive molecules, such as sphingosine, ceramides, phosphosphingolipids, and glycosphingolipids, which are the main structural lipids of various membranes in nervous tissue. Lipids have other important biological functions such as: messengers in signal transduction, for example, steroid hormones which can influence brain function^[73]^; modulators for enzyme activity, for example, lecithin, which can activate β-hydroxybutyrate dehydrogenase^[74]^; precursors of hormones, vitamins, growth factors, and antioxidants; and mediators of signal recognition and immunity, for example, glycolipids^[75]^. The intestinal microbiota is also pivotal for the biosynthesis of hepatic membrane phospholipids and liver regeneration^[76]^. Gut microbiome-derived lipids can influence the function of brain and liver through the signaling pathway mediated by macrophageinducible Ctype lectin, spleen tyrosine kinase, and nuclear factor kappa-B^[77-79]^. LPS, a bacteria-derived lipid, can enter the bloodstream when the gut barrier function is impaired and the permeability of the barrier is increased, to induce inflammation by activating the toll-like receptors (TLRs)^[80]^. Steroid hormones have been reported to have the ability to regulate the metabolism of sphingolipids, which can inversely regulate the secretion of steroid hormones^[81]^. Additionally, BAs can regulate lipid metabolism via the FXR and TGR5 pathways which are linked to the gut-liver-brain axis^[82]^. Abnormal lipid accumulation may cause impairments of the brain^[83]^ and the liver^[84]^. The liver is also implicated in shaping the gut microbiome and lipid metabolism^[85]^. Therefore, lipid metabolism is involved in the physiological functions of organs such as the brain, gut, and liver, while dysregulation of lipid metabolism can affect the structure and function of the gut microbiome.

### Uric acid

Uric acid (UA), a scavenger of oxygen radical, is synthesized mainly in the liver, intestines, and the vascular endothelium as the end metabolite of purine, and excreted via the kidney/intestine. UA is involved in disorders of not only the kidney^[86]^, but also the liver^[87]^, the joint^[88]^, the brain^[89]^, and the cardiovascular system^[90]^ via several mechanisms including oxidative stress, inflammation, and apoptosis. Well-known causes of serum hyperuricemia include UA metabolism disturbance and reduction of its secretion by kidney. Many intestinal bacteria, including species that belong to the genus *Micrococcus*, *Streptomyces*, *Pseudomonas*, and *Bacillus*, carry genes coding for uricase/urate oxidase which can degrade dietary and endogenous uric acid^[91]^. Intestinal *Akkermansia muciniphila* exhibited beneficial effects of decreasing serum urate and inhibiting xanthine oxidase in the liver^[92]^. Additionally, recent studies have identified that a dysbiosis of intestinal microbiota underlies the pathological association between serum UA and body mass index (BMI)^[93]^. UA has been implicated in the pathophysiology of liver damage^[94]^ and cognitive decline in attention and executive function^[95]^. Accumulating case studies and experiments in animal models have provided mechanistic insights to explain the complicated pathophysiology of UA-associated disorders. UA has been reported to have neuroprotective properties by activating the nuclear factor E2 related factor 2 (Nrf2) pathway including γ-glutamate-cysteine ligase catalytic subunit (γ-GCLC), heme oxygenase-1 (HO-1), and NQO1^[96]^. However, hyperuricemia can cause chronic inflammation by activating inflammatory mediators such as TLR- 4 and the inflammatory vesicles of the Pyrin domain NOD-like receptor family 3 (NLRP3)^[97,98]^. UA can also downregulate NO levels by enhancing arginase activity, thereby deteriorating pulmonary arterial hypertension^[99]^. We speculate that this may deteriorate portal hypertension in cirrhotic patients to contribute to the progression of HE. Further research is needed in humans to identify UA-mediated pathways that are involved in the regulation of the microbiota-gut-liver-brain axis in order to find a strategy to reduce the risks of HE.

## IMPLICATIONS OF THE MICROBIOME-GUT-LIVER-BRAIN AXIS IN EARLY WARNING, DIAGNOSIS, AND TREATMENT OF HEPATIC ENCEPHALOPATHY

Identification of high-risk cohorts, accurate diagnosis of HE, and timely interventions are all important arms of the strategy to prevent and treat HE. During the progression of liver and neurological disease, the communication networks are complex, and these networks involve both molecular communication between organs and systems within the host, as well as communication between gut microbiota-derived metabolites and the host. Furthermore, there is also the complex interaction between the host nervous and immune system which includes neurotransmitters, interleukins, chemotactic factors, tumor necrosis factor, colony-stimulating factor, interferons, other cytokines such as transforming growth factor β, vascular endothelial cell growth factor, and many more. The high level of complexity suggests that uncovering the mechanisms of various diseases will require high sensitivity/high-throughput technologies. With the recent introduction of multi-omics approach into disease-associated microbiome studies, delineating the networks involved in the pathogenesis of liver disease may identify key nodes that could be the targets for risk assessment of HE progression, early warning signs, evaluation of treatment prognosis, and therapeutic strategies to prevent disease complications and symptom progression.

### The microbiome-gut-liver-brain axis as predictive and diagnosis tools for hepatic encephalopathy

Since the progression of HE, from the development of the disease to more advanced stages of the disease, is inevitably accompanied by alterations in the structure and function of the microbiome and the metabolites, it is likely that there are specific microbial genes and metabolites from the microbiome that can be used as biomarkers for early warning and diagnosis of HE. Many studies have been performed to explore and identify diagnostic indicators of HE in the clinical laboratory. The gut microbiome was first considered as a unique non-invasive diagnostic biomarker for early- and late-stage liver cancer. This idea has been validated by a study in a cross-regional cohort^[26]^, which indicated that the diagnostic model for liver diseases using microbiome was proven to be satisfactory in multi-regional Chinese populations. Promising gut microbiota indicators to differentiate patients with higher HE progression risk from cirrhotic cohorts included: high populations of urease enzyme-producing bacteria such as *Alcaligenaceae*, *Streptococcaceae*, and *Staphylococcus intermedius* in fecal microbiota^[100,101]^; low populations of butyric acid-producing bacteria such as *Faecalibacterium prausnitzii* in colonic mucosal samples; and low populations of *Ruminococcus*, *Clostridium XIVb*, *Faecalibaterium*, and *Butyricoccus* in stools^[102-104]^. In addition, α-ketoglutaramate, a metabolite in the process of glutamine transamination, has been proposed to be a useful biomarker for early prediction of HE^[105]^. Serum homocysteine and BA levels were also identified as promising markers for the diagnosis of HE^[106]^. Additionally, it is possible to target brain dysfunction as a potential diagnostic criterion in HE, as the brain default mode network based on resting-state functional magnetic resonance imaging (MRI) scanning was shown to be a valuable method to identify HE^[107]^. All of these diagnostic indicators for clinical laboratory are closely related to the microbiome-gut-liver-brain axis.

### The microbiome-gut-liver-brain axis as therapeutic and preventive targets for hepatic encephalopathy

At present, HE therapies targeting the brain are still in the preclinical stage^[108]^. For instance, gamma-aminobutyric acid-benzodiazepine receptor agonists reduced peripheral inflammation and neuroinflammation which was associated with improved cognitive and motor functions in hyperammonemic rats^[109]^. The principles of HE prevention and treatment emphasize the importance of reducing and eliminating neurological, neuropsychiatric, and motor complications by eliminating triggers and reducing the production and absorption of intestinal toxins. A number of clinical trials have been conducted to explore possible treatments for HE^[110]^. Currently, the main therapeutic strategies include temporarily reducing or even prohibiting protein intake^[111]^, oral administration of weakly acidic solutions, for example, dilute acetic acid for catharsis^[112]^, microbiota-targeted interventions such as lactulose, probiotics, rifaximin, and FMT to reshape the gut microbiome to reduce the production of toxins^[113-116]^, glutamic acid tablets to promote free ammonia conversion^[117]^, arginine to promote urea synthesis^[118]^, ornithine to lower blood ammonia and correct symptoms of amino acid metabolism disorder^[119]^, branched-chain amino acids to suppress the formation of pseudo-neurotransmitters^[120]^, naloxone or other opioid receptor antagonists to improve brain dysfunction syndrome^[121]^, and the use of an artificial liver support system to eliminate all triggers^[122]^. These therapeutic strategies are included in the guidelines on the diagnosis and treatment of HE^[4]^. Of these, microbiota and metabolomic-targeted strategies, in particular lactulose, rifaximin, and probiotics^[118]^, are well-received in the clinic for the treatment of covert/minimal HE and to prevent HE reoccurrence^[123]^. FMT, a newer modality of therapies targeting the gut microbiome that has come into practice in the past decade, has been demonstrated to be safe and effective in preliminary clinical trials on a small number of patients with HE^[124]^. Before FMT, many procedures had to be strictly performed to prevent the occurrence of adverse events, including selection of donors by questionnaire, previous medical records, physical and stool examinations, evaluations of the potential risks and benefits for the recipients in clinical situations, postoperative risks, *etc*., and preparations of the raw material in the super-clean room under close microbiological control. After FMT, medical observation and periodic follow-ups should be taken to monitor the clinical efficacy and short- and long-term adverse events, such as mild and self-limiting abdominal discomfort, cramping, bloating, diarrhea or constipation, and, rarely, the transmission of diseases that cannot be tested for by screening^[125]^. However, there are still many challenges that remain to be overcome clinically for personalized HE interventions that target the microbiota-gut-liver-brain axis. For example, challenges include: how to accurately modify the gut microbiome and its metabolites to give predictable clinical outcomes; how to maintain these outcomes; and how to anticipate endogenous risks after the interventions. Randomized placebo-controlled multi-center trials on a large cohort should be performed to validate the clinical outcomes of these preventive care interventions to prevent symptoms in HE patients from progressing to severe neuropsychiatric dysfunction, such as coma.

## CONCLUSION

As mentioned above, research on the microbiome-gut-brain axis and the microbiome-gut-liver axis has proved that the gut microbiome is not only involved in the process of liver disease exacerbation, but also closely related to cognitive impairment. These studies have allowed us to further understand the microbiota-brain-gut-liver axis, providing new strategies to prevent and treat HE. While these previous studies focused on identifying the differences in the amounts of microbes and small molecule metabolites between HE patients and the control group, the exact dynamics of molecular networks that are involved in gut microbiota-liver-brain axis in the context of HE disease progression from covert HE to coma is still unclear. In addition, although the clinical efficacy of intestinal microbiota reconstruction therapy for HE, including probiotics, prebiotics, and FMT, has been recognized, there is still a lack of strong biomarkers that can be used to monitor the efficacy in real time and evaluate the prognosis.

## DECLARATIONS

### Copyright

© The Author(s) 2024.
